# Compensatory Mechanism of Maintaining the Sagittal Balance in Degenerative Lumbar Scoliosis Patients with Different Pelvic Incidence

**DOI:** 10.1111/os.12805

**Published:** 2020-09-21

**Authors:** Chao Liu, Fan‐qi Hu, Wen‐hao Hu, Kai Song, Gen‐long Jiao, Guo‐quan Zheng, Xue‐song Zhang, Zhi‐zhong Li

**Affiliations:** ^1^ Department of Orthopaedics The First Affiliated Hospital of Jinan University Guangzhou China; ^2^ Spine Division, Department of Orthopaedics, The First Medical Center Chinese People's Liberation Army General Hospital Beijing China; ^3^ Department of Orthopaedics, the Fourth Medical Center Chinese People's Liberation Army General Hospital Beijing China

**Keywords:** Pelvic incidence, Sagittal balance, Degenerative lumbar scoliosis

## Abstract

**Objective:**

To investigate the compensatory mechanism of maintaining the sagittal balance in degenerative lumbar scoliosis patients with different pelvic incidence (PI).

**Methods:**

This was a retrospective imaging observation study. Patients in our department with degenerative lumbar scoliosis between 2017 and 2019 were reviewed. A total of 36 patients were eligible and included in the present study. The average age of those patients was 64.22 years, including 8 men and 28 women. The coronal and sagittal parameters were measured on full‐length spine X‐ray film, including globe kyphosis (GK), lumber lordosis (LL), thoracolumbar kyphosis (TLK), thoracic kyphosis (TK), sagittal vertical axis (SVA), sagittal shift angle, Cobb angle, coronal shift angle, and vertebra. The anterior pelvic plane angle (APPA) and pelvic parameters were also measured, including the pelvic tilt (PT), the PI, and the sacral slope (SS). PI‐LL, LL‐SS, and GK‐SS were calculated. Traditional pelvic tilt was also calculated using the following formula: cPT = PI × 0.37–7. These patients were divided into two groups according to their PI values. The patients’ PI value in Group 1 was smaller than 50°. The patients’ PI value in Group 2 was equal to or larger than 50°.

**Results:**

These patients’ SS, PT, PI, LL, TLK, TK, and GK were 28.70° ± 11.36°, 23.28° ± 6.55°, 52.00° ± 11.03°, 31.66° ± 14.12°, 12.12° ± 14.9°, 17.81° ± 13.53°, and −13.17° ± 16.27°. The sagittal shift angle, the APPA, the Cobb angle, the coronal shift angle, vertebra, PI‐LL, cPT, APPA‐4, LL‐SS, and GK‐SS were 4.38° ± 5.75°, −12.55° ± 8.83°, 30.03° ± 12.59°, 2.40° ± 2.13°, 4.08 ± 0.93, 19.86° ± 10.97°, 12.35° ± 4.55°, −8.30° ± 9.07°, 3.30° ± 8.82°, and 15.53° ± 9.83°, respectively. There was no significant difference between PT and cPT + APPA‐4 or between cPT and PT‐APPA+4. There was significant difference between PT and cPT + APPA or between cPT and PT‐APPA. This demonstrated that the APPA‐4 is reliable as degree of the pelvic sagittal retroversion. There were significant differences in SS, PI, LL, TLK, GK, APPA, PT‐APPA, PT‐APPA+4, cPT, and APPA‐4 between Group 1 and Group 2. There were no significant differences in PT, TK, sagittal shift angle, SVA, Cobb angle, coronal shift angle, vertebra number, PI‐LL, cPT + APPA, cPT + APPA‐4, LL‐SS, and GK‐SS between Group 1 and Group 2. The Pearson tests showed that PI‐LL had significant correlations with TK, LL, sagittal shift angle, SVA, and LL‐SS. There was no significant correlation between PI‐LL and Cobb angle, GK, TLK, APPA, vertebra, Coronal Shift Angle, or GK‐SS.

**Conclusion:**

The APPA‐4 is reliable as degree of the pelvic sagittal retroversion. In degenerative lumbar scoliosis, patients with smaller PI tended to rely more on the pelvic retroversion to maintain the sagittal balance than patients with larger PI, or patients with smaller PI were likely to start up the pelvic retroversion compensatory mechanism earlier than the patients with larger PI.

## Graphical abstract

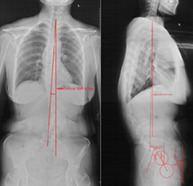

On the anterior–posterior, full‐length spine X‐ray film, the coronal shift angle was the angle formed by the C_7_ plumb line and the line through the seventh cervical spinous process and the spinous process of the first sacrum vertebra. The sagittal shift angle is the angle formed by the C_7_ plumb line and the line through the seventh cervical vertebra center and the superior–posterior corner of the first sacrum vertebra on the lateral full‐length spine X‐ray film. APPA is the angle formed by the anterior pelvic plane (APP) and the vertical line. PI, pelvic incidence; PT, pelvic tilt; SS, sacral slope.

## Introduction

Degenerative lumbar scoliosis (DLS) is a spinal three‐dimensional deformity with coronal deviation of greater than 10°. Degenerative scoliosis is the result of progressive and asymmetric degeneration of the disk and facet joints, which lead to spinal column malalignment^1^. Degenerative lumbar scoliosis is generally associated with back pain, lower‐limb pain, and functional impairment, and usually occurs in the aging population^2^. With an aging population worldwide, increasing attention is being paid to the quality of life in the elderly population. Degenerative scoliosis has become a considerable healthcare concern^3^. The prevalence of degenerative scoliosis has been reported as ranging from 6% to 68% in the elderly population^1^. Degenerative scoliosis is a form of adult scoliosis. Adult scoliosis is a collective term comprising all spinal deformities in the adult individual^1^. Compared with adult idiopathic scoliosis, DLS involves fewer segments and less severe curves^2^. Coronal imbalance is the main cause of back pain, poor health‐related quality of life, and unsatisfactory appearance in these patients^4^. Sagittal imbalance has an important impact on patients’ health status and is closely connected to the health‐related quality of life of patients with DLS^1^, ^5^. The degree of sagittal imbalance is closely related to the severity of symptoms and functional status^6^, ^7^. Daubs *et al*. reported that after surgery, the Oswestry disability index (ODI) score could achieve a 0.395‐point improvement with each 1‐mm sagittal imbalance correction, and the change in sagittal imbalance also had significant effects on the Scoliosis Research Society scores^8^. Schwab *et al*. reported that patients with more severe sagittal imbalance were more prone to receive surgical intervention for restoring spine alignment^9^. Patients with more severe sagittal imbalance tended to have worse postoperative symptoms, disability, and operative complications^10^, ^11^. Patients with more severe sagittal imbalance had higher adjacent segment disease and non‐union and revision surgery rates, and worse SF‐36 and ODI scores^10^. Complication rates, such as for junctional kyphosis, pseudoarthrosis, adjacent segment degeneration, and implant‐related complications, were higher in patients with severe sagittal imbalance^11^.

When trunk sagittal imbalance occurs in patients, the pelvic retroversion is also a compensatory factor besides the lower extremity joints compensatory mechanism for maintaining the patients’ globe sagittal balance. Hence, studying the sagittal alignment of the spine and the pelvis of patients with DLS is critical for making a surgical plan, and the analysis of sagittal spino–pelvic alignment in DLS is necessary. Awareness of the pelvic parameters is essential to understand patients’ sagittal balance and implement the best surgical strategy in patients with degenerative spine diseases. The pelvic parameters include pelvic incidence (PI), pelvic tilt (PT), and sacral slope (SS). The PI is widely accepted as a constant pelvic parameter in adulthood, which does not rely on the position of the pelvis^12^. In patients with kyphosis deformity, sagittal compensation potential greatly depends on the PI: lower PI means much less compensation potential and higher PI means greater compensation potential^12^. Han *et al*. reported that high PI may have an impact on the pathogenesis of DLS^13^. There are also several published studies examining the influence of PI and spine–pelvic mismatch (PI‐LL) on surgical clinical outcomes^14^, ^15^. However, fewer studies have investigated how patients with different PI maintain their sagittal balance before surgery.

The anterior pelvic plane (APP) is widely used by joint surgeons as an anatomical reference plane of the pelvis during the procedure of total hip replacement and is also commonly accepted as the coronal plane of the pelvis^16^, ^17^, ^18^. The APP is the plane formed by both anterior superior iliac spines and the pubic symphysis^19^. However, the APP is not the real coronal plane of the pelvis; the angle between the APP and the vertical line (APPA) is approximately 4° when normal subjects are in their natural standing position^20^. The APPA was defined as the angle between the line connecting the midpoint of both anterior superior iliac spines to the pubic symphysis and the vertical line of the lateral radiograph of the pelvis in the standing position^19^. Therefore, the APPA minus 4° should be equal to the degree of pelvic retroversion. The measured PT value minus the degree of pelvic retroversion should be the PT value that the patients had before they had sagittal imbalance. According to the study conducted by Vialle *et al*., PT was calculated using the following formula: cPT = PI×0.37–7^21^. PT(cPT) was calculated according to the PI. If the APPA‐4 represents the pelvic retroversion degree, cPT plus APPA and minus 4° should be equal to the measured PT, and *vice versa*.

We hypothesized that the APPA‐4 could represent the pelvic sagittal retroversion degree, and there was difference in the compensatory mechanism of maintaining the sagittal balance in DLS patients with different PI. Hence, the purpose of this study was to investigate: (i) whether the APPA‐4 is reliable as degree of the pelvic sagittal retroversion; and (ii) the compensatory mechanism of maintaining the sagittal balance in DLS patients with different PI.

## Materials and Methods

### *Patients*

All materials were used with the consent of patients. This study was approved by the medical ethics committee of our institution. Patients in our department with DLS between 2017 and 2019 were reviewed.

#### *Inclusion and Exclusion Criteria*

Inclusion criteria: (i) patients were diagnosed with lumbar degenerative scoliosis; (ii) before surgery, patients had undergone anterior–posterior and lateral full‐length spine X‐ray test; (iii) the lateral full‐length spine X‐ray film included the total pelvis; (iv) patients had not had previous pelvic trauma, surgical history, or congenital pelvic and spinal diseases; and (v) this study was a retrospective and observational study.

Exclusion criteria: (i) lateral full‐length spine X‐ray film did not include the full pelvis; (ii) patients with previous pelvic trauma and surgery history; and (iii) patients with congenital pelvic and spine diseases.

A total of 36 patients were eligible and included in this present study. The average age of patients was 64.22 years, with 8 men and 28 women included.

### *Radiographic Parameters*

All the parameters were measured twice, and the averages were adopted.

#### *Sagittal Parameters*

**Fig. 1:**
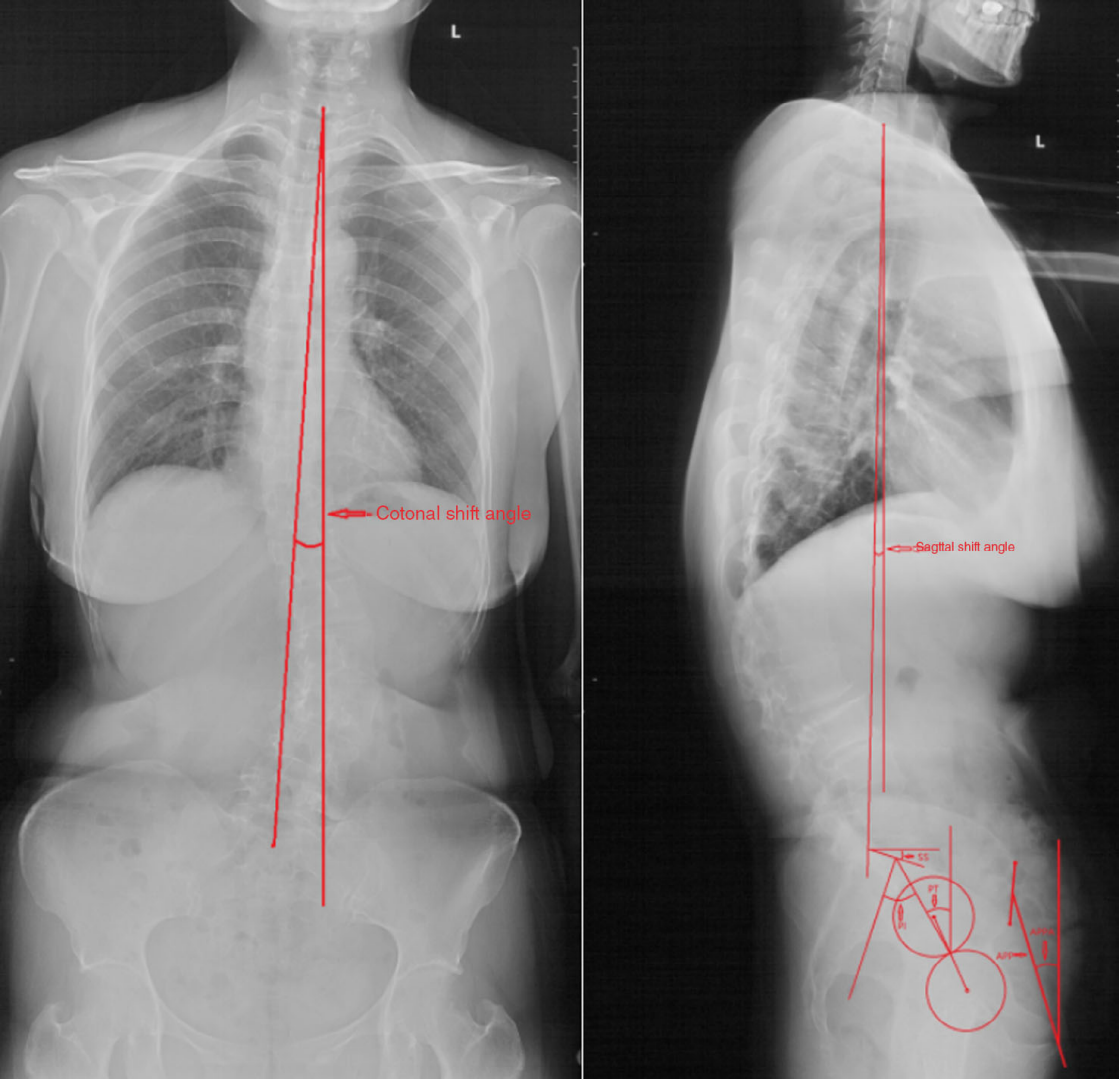
On the anterior–posterior full‐length spine X‐ray film, the coronal shift angle was the angle formed by the C_7_ plumb line and the line through the seventh cervical spinous process and the spinous process of the first sacrum vertebra. The sagittal shift angle was the angle formed by the C_7_ plumb line and the line through the seventh cervical vertebra center and the superior–posterior corner of the first sacrum vertebra on the lateral full‐length spine X‐ray film. APP, anterior pelvic plane; APPA; anterior pelvic plane angle; PI, pelvic incidence; PT, pelvic tilt; SS, sacral slope.

The globe kyphosis (GK), lumber lordosis (LL), thoracolumbar kyphosis (TLK), thoracic kyphosis (TK), and sagittal vertical axis (SVA) were measured. The GK was commonly used to represent the patient's trunk sagittal imbalance degree. The LL, TLK, and TK are widely used to reflect the changes in the physiological curve of the spine (Fig. 3). The GK was measured from the superior end plate of the T_5_ thoracic vertebra to the superior end plate of the S_1_ vertebra. LL was defined as the Cobb angle between the two lines parallel to the superior endplate of L_1_ and the S_1_ vertebra, respectively; TLK was defined as the Cobb angle between the two lines parallel to the superior endplate of T_11_ and the superior endplate of L_2_, respectively; TK was defined as the Cobb angle between the two lines parallel to the superior endplate of T_5_ and the superior endplate of L_1_, respectively. The trunk sagittal shift angle, the angle formed by the C_7_ plumb line and the line through the seventh cervical vertebra center and the superior–posterior corner of the first sacrum vertebra, was also measured on the lateral full‐length spine X‐ray film (Fig. 1). The sagittal shift angle also represents the degree of patients’ sagittal imbalance.

#### *Coronal Parameters*

The scoliosis Cobb angle was measured on the anterior–posterior full‐length spine X‐ray film. The trunk coronal shift angle, the angle formed by the C_7_ plumb line and the line through the seventh cervical spinous process and the spinous process of the first sacrum vertebra, was also measured on the anterior–posterior full‐length spine X‐ray film (Fig. 1). The coronal shift angle represents the degree of coronal trunk deviation. The number of the vertebra included in the scoliosis was recorded.

#### *Pelvic Parameters*

**Fig. 2:**
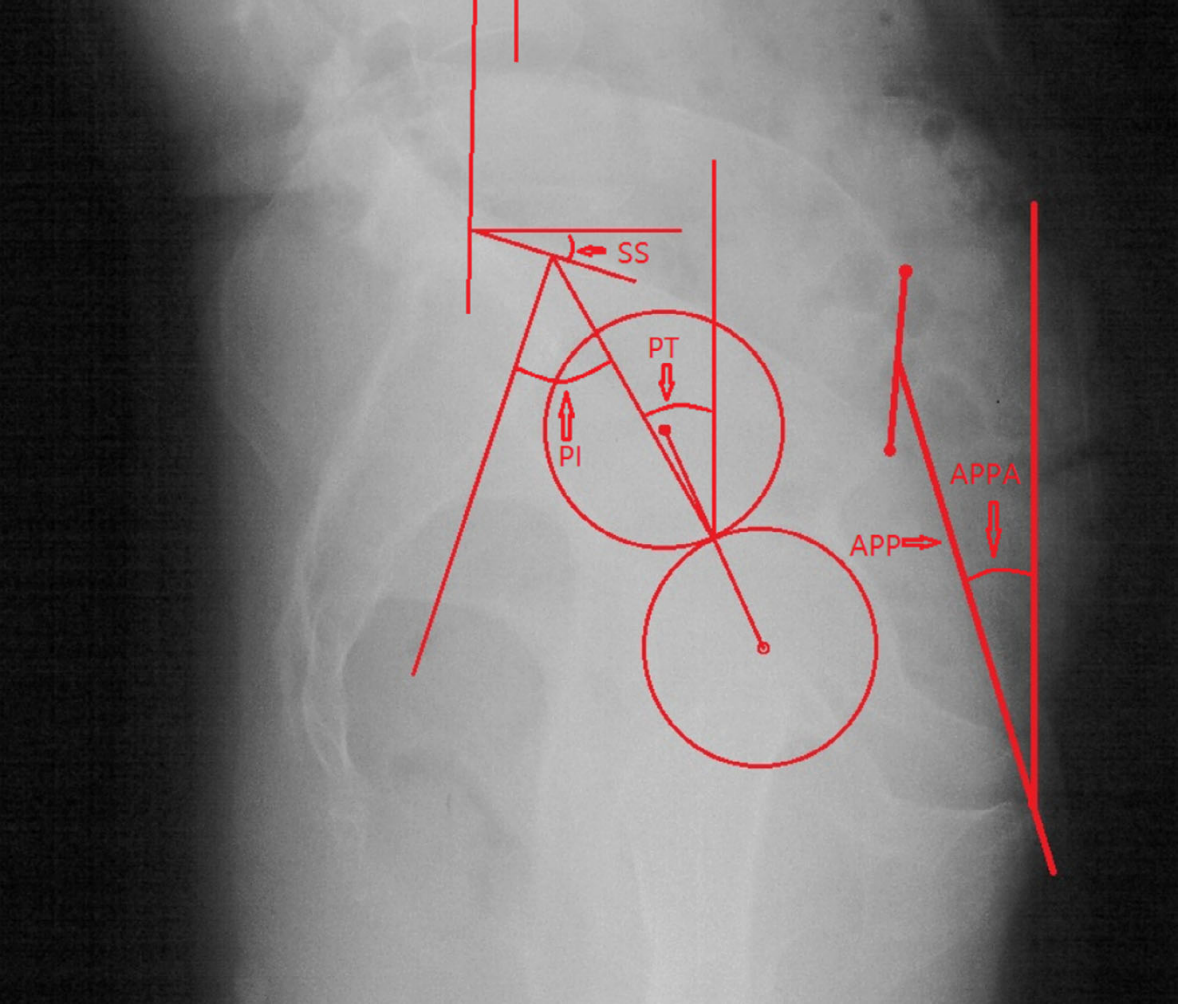
The anterior pelvic plane (APP) was the plane formed by both anterior superior iliac spines and the pubic symphysis. APPA was defined as the angle between the line connecting the midpoint of both anterior superior iliac spines to the pubic symphysis and the vertical line of the lateral radiograph of the pelvis in the standing position. Pelvic incidence (PI) is the angle formed by the line connecting the midpoint of the bilateral femoral head center point to the center point of the sacral endplate and the line perpendicular to the sacral endplate. Pelvic tilt (PT) is the angle between the line connecting the midpoint of the bilateral femoral head center point to the center point of the sacral endplate and the vertical line; the sacral slope (SS) is the angle between the superior plate of S_1_ and the horizontal line.

The PT, PI, and SS were measured on the pelvic lateral X‐ray film. The pelvic incidence (PI) is the angle formed by the line connecting the midpoint of bilateral femoral head center point to the center point of the sacral endplate and the line perpendicular to sacral endplate. The pelvic tilt (PT) is the angle between the line connecting the midpoint of the bilateral femoral head center point to the center point of the sacral endplate and the vertical line. The sacral slope (SS) is the angle between the superior plate of S1 and the horizontal line (Fig. 2). APP is the plane formed by both anterior superior iliac spines and the pubic symphysis^19^. APPA is defined as the angle between the line connecting the midpoint of both anterior superior iliac spines to the pubic symphysis and the vertical line of the lateral radiograph of the pelvis in the standing position^19^. The APPA was also measured on the pelvic lateral X‐ray film (Fig. 2). Because the angle between the APP and the vertical line was approximately 4° when normal subjects were standing in their natural standing position^20^, the APPA was adjusted by subtracting 4 to represent the pelvic retroversion degree. The calculated PT (cPT) was obtained according to the formula cPT = PI×0.37–7^21^. Because the LL included the SS, the LL‐SS was calculated to eliminate the influence of SS on the LL. Because the GK also included the SS, the GK‐SS was calculated to eliminate the influence of SS on the GK. PI‐LL was also obtained.

**Fig. 3:**
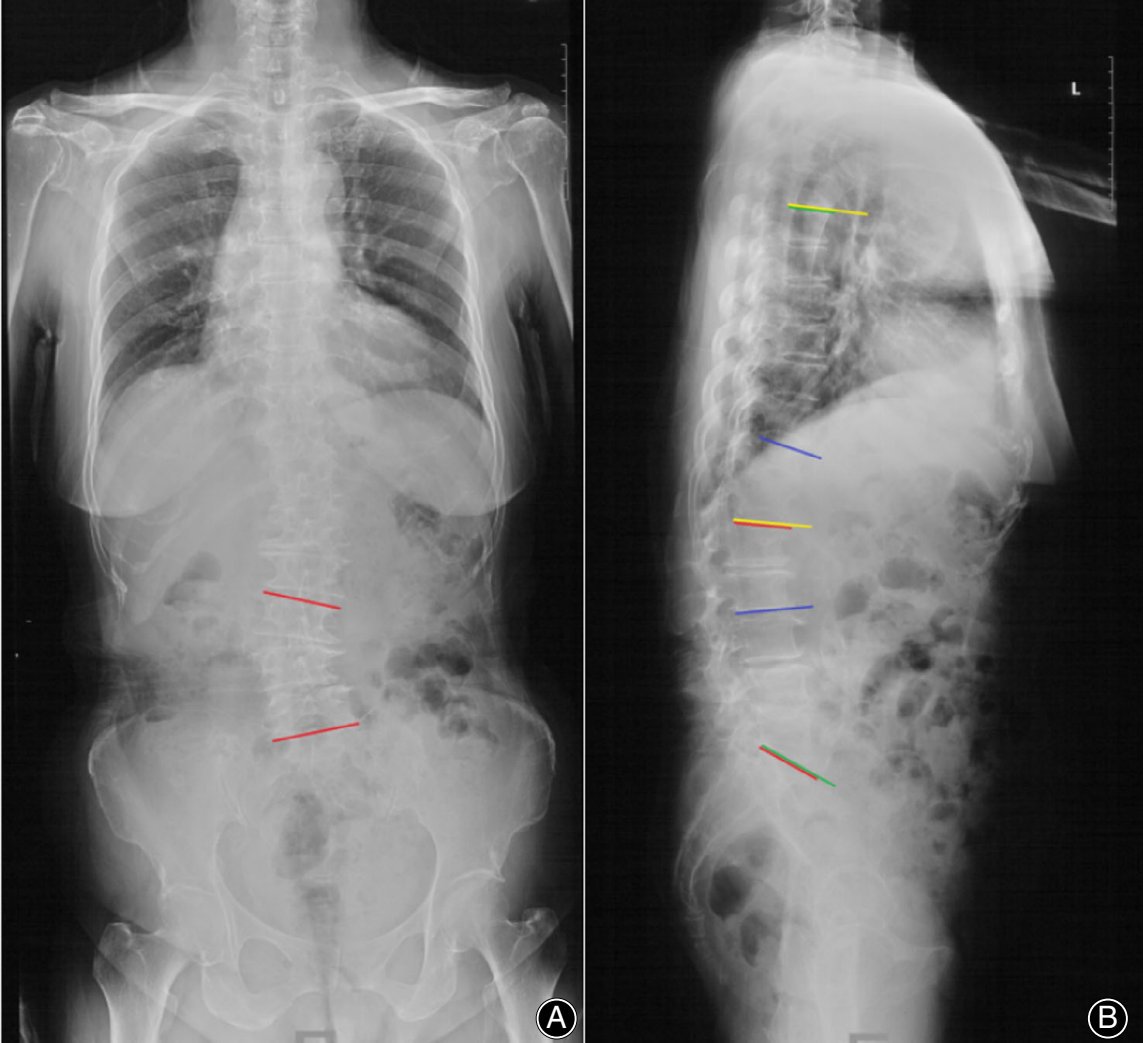
In fig3A, the scoliosis Cobb angle was formed by the red lines. In fig3B, GK was measured from the superior end plate of the T_5_ thoracic vertebra to the superior end plate of the S_1_ vertebra(angle formed by the green lines). LL was defined as the Cobb angle between the two lines parallel to the superior endplate of L_1_ and the S_1_, respectively(angle formed by the red lines); TLK was defined as the Cobb angle between the two lines parallel to the superior endplate of T_11_ and the lower endplate of L_2_, respectively (angle formed by the blue lines); TK was defined as the Cobb angle between the two lines parallel to the superior endplate of T_5_ and the superior endplate of L_1_, respectively(angle formed by the yellow lines).

### *Group of Patients*

A normal PI mean value ranges from 50° to 55°^22^. Patients were divided into two groups according to patients’ PI. The patients’ PI in Group 1 was smaller than 50° (13 patients, 36.1%). The patients’ PI in Group 2 was equal to or larger than 50° (23 patients, 63.9%).

### *Statistical Analysis*

Data analysis was performed using SPSS version 23.0 for Windows. All the data was presented as mean ± standard deviation. For testing the reliability of APPA‐4 as pelvic retroversion degree, the cPT + APPA‐4 was compared with measured PT, and the PT‐APPA+4 was also compared with cPT *via* a paired sample *t*‐test. Because some authors adopt APP as the coronal plane of the pelvis, a paired sample *t*‐test was also performed to determine the difference between the PT and cPT + APPA and between cPT and PT‐APPA. An independent *t*‐test was performed to determine the differences between the two groups. A Pearson test was performed to find the correlation between the PI‐LL and other parameters. A *P*‐value less than 0.05 was considered significant in all analyses.

## Results

### *Radiographic Characteristics of All Patients*

These patients’ SS, PT, PI, LL, TLK, TK, and GK were 28.70° ± 11.36°, 23.28° ± 6.55°, 52.00° ± 11.03°, 31.66° ± 14.12°, 12.12° ± 14.9°, 17.81° ± 13.53°, and − 13.17° ± 16.27°. The sagittal shift angle, APPA, Cobb angle, coronal shift angle, vertebra number, PI‐LL, cPT, APPA‐4, LL‐SS, and GK‐SS were 4.38° ± 5.75°, −12.55° ± 8.83°, 30.03° ± 12.59°, 2.40° ± 2.13°, 4.08 ± 0.93, 19.86° ± 10.97°, 12.35° ± 4.55°, −8.30° ± 9.07°, 3.30° ± 8.82°, and 15.53° ± 9.83°, respectively.

### *Analysis of the Reliability of* *APPA‐4 as Pelvic Retroversion Degree*

There was no significant difference between the PT and cPT + APPA‐4 (23.28° ± 6.55°, 21.82° ± 7.33°; *P* = 0.137). There was also no significant difference between the cPT and PT‐APPA+4 (12.35° ± 4.55°, 14.19° ± 7.70°; *P* = 0.087). Therefore, the APPA‐4 is reliable as degree of the pelvic sagittal retroversion. There was significant difference between PT and cPT + APPA (23.28° ± 6.55°, 25.82° ± 7.33°; *P* = 0.012) or between cPT and PT‐APPA (12.35° ± 4.55°, 10.19° ± 7.70°; *P* = 0.046).

### *Comparison between the Two Groups*

The results showed that there was significant difference in SS, PI, LL, TLK, GK, APPA, PT‐APPA, PT‐APPA+4, cPT, and APPA‐4 between Group 1 and Group 2 (Table 1). The results revealed that there was no significant difference in PT, TK, sagittal shift angle, SVA, Cobb angle, coronal shift angle, vertebra number, PI‐LL, cPT + APPA, cPT + APPA‐4, LL‐SS, and GK‐SS between Group 1 and Group 2 (Table 1) .

**TABLE 1:** Comparison between Group 1 and Group 2 (mean ± standard deviation)

Parameters	Group 1	Group 2	*P‐*value
Age (years)	62.30 ± 7.96	66.13 ± 7.53	0.171
SS (°)	17.43 ± 6.48	35.08 ± 8.05	0.000
PT (°)	23.14 ± 4.72	23.36 ± 7.49	0.925
PI (°)	40.90 ± 5.81	58.28 ± 7.82	0.000
LL (°)	21.41 ± 8.60	37.45 ± 13.41	0.000
TLK (°)	23.50 ± 14.62	5.70 ± 10.83	0.000
TK (°)	21.66 ± 14.62	15.63 ± 11.58	0.204
GK (°)	2.43 ± 13.55	−21.99 ± 9.79	0.000
Sagittal shift angle (°)	4.79 ± 4.65	4.15 ± 6.38	0.755
SVA (mm)	67.42 ± 53.55	56.46 ± 53.70	0.589
APPA (°)	−18.06 ± 7.29	−9.43 ± 8.18	0.003
Cobb angle (°)	31.18 ± 11.70	29.39 ± 13.28	0.688
Coronal shift angle (°)	2.16 ± 1.52	2.54 ± 2.43	0.614
Vertebra	4.07 ± 1.11	4.08 ± 0.84	0.976
PI‐LL (°)	17.94 ± 7.70	20.95 ± 12.48	0.437
PT‐APPA (°)	5.09 ± 4.83	13.08 ± 7.59	0.002
PT‐APPA+4 (°)	9.09 ± 4.83	17.08 ± 7.59	0.002
cPT (°)	8.13 ± 2.14	14.74 ± 3.74	0.000
APPA‐4 (°)	−14.06 ± 7.29	−5.43 ± 8.18	0.003
cPT + APPA (°)	26.20 ± 8.56	25.60 ± 6.75	0.818
cPT + APPA‐4 (°)	22.20 ± 8.56	21.60 ± 6.75	0.830
LL‐SS (°)	4.93 ± 6.44	2.37 ± 9.94	0.411
GK‐SS (°)	19.86 ± 12.72	13.08 ± 6.94	0.094

The sagittal shift angle is the angle formed by C_7_ plumb line and the line through the seventh cervical vertebra center and the superior‐posterior corner of the first sacrum vertebra on the lateral full‐length spine X‐ray film. APPA is the angle between the anterior pelvic plane (APP) and the vertical line. The Cobb angle is the degenerative lumbar scoliosis Cobb angle. The coronal shift angle is the angle formed by the C_7_ plumb line and the line through the seventh cervical spinous process and the spinous process of the first sacrum vertebra on the anterior–posterior full‐length spine X‐ray film. cPT: The calculated PT according to the formula PT = PI×0.37–7. APPA‐4: Because APPA is −4° when normal subjects were standing in their natural standing position, the APPA was adjusted by subtracting 4 for representing pelvic retroversion degree.

GK, globe kyphosis; LL, lumber lordosis; SS, sacral slope; SVA, sagittal vertical axis; TK, thoracic kyphosis; TLK, thoracolumbar kyphosis; GK‐SS, GK minus SS; LL‐SS, LL minus SS.

### *Correlations between* *PI‐LL* *and Other Parameters*

The Pearson tests showed that PI‐LL had significant correlations with TK, LL, sagittal shift angle, SVA, and LL‐SS, respectively (Table 2). There was no significant correlation between PI‐LL and Cobb angle, GK, TLK, APPA, vertebra, coronal shift angle, and GK‐SS (Table 2) .

**TABLE 2:** Correlations between PI‐LL and other parameters

Parameters	*r‐*value	*P*‐value
Cobb angle	0.081	0.640
GK	0.022	0.899
TK	−0.549	0.001
TLK	0.087	0.615
LL	−0.578	0.000
APPA	−0.316	0.060
Vertebra	0.139	0.417
Coronal shift angle	0.084	0.627
Sagittal shift angle	0.470	0.004
SVA	0.540	0.000
LL‐SS	−0.775	0.000
GK‐SS	−0.086	0.619

APPA, anterior pelvic plane angle; GK, globe kyphosis; LL, lumber lordosis; SS, sacral slope; SVA, sagittal vertical axis; TK, thoracic kyphosis; TLK, thoracolumbar kyphosis; GK‐SS, GK minus SS; LL‐SS, LL minus SS; PI‐LL, PI and spine–pelvic mismatch.

## Discussion

### *Reliability of* *APP‐4 as the Pelvic Sagittal Retroversion Degree*

Degenerative lumbar scoliosis is a degenerative deformity of the spine with a scoliosis Cobb angle larger than 10°. The incidence of DLS is reported to be more than 60% in the elderly^1^. In the current aging society, DLS has become an increasing healthcare issue in the elderly. In DLS, the sagittal imbalance has an important impact on patients’ health status^1^, ^5^. Pelvic retroversion is one of the compensatory mechanisms to maintain sagittal balance in patients with DLS. APP is commonly accepted as the coronal plane of the pelvis^16^, ^17^, ^18^. However, some authors report that the angle between the APP and the vertical line (APPA) is approximately 4° when normal subjects are in their natural standing position^20^. In theory, the APPA minus 4° equals the degree of pelvic retroversion. In this study, we also tested the reliability of APPA‐4 as pelvic retroversion degree. The PT that the patients should have had before they had sagittal imbalance was calculated using the following formula: PT = PI×0.37–7^21^. We compared cPT + APPA‐4 with the PT and compared the PT‐APPA+4 with cPT. The cPT + APPA was also compared with PT. PT‐APPA was compared with cPT. The statistical results showed that there was no significant difference between cPT + APPA‐4 and PT or between PT‐APPA+4 and cPT. There was significant difference between cPT + APPA and PT and between PT‐APPA and cPT. This suggests that the APP‐4 reliably reflects the degree of the pelvic sagittal retroversion. Consistent with previous studies, the results also demonstrated that the APP was not the pelvic coronal plane.

### *Compensatory Mechanism of Maintaining the Sagittal Balance in Degenerative Lumbar Scoliosis Patients with Different Pelvic Incidence*

Understanding spine sagittal balance is a primordial factor in producing an accurate surgical plan for DLS patients to enable a good clinical outcome^12^. Since Duval‐Beaupere *et al*.^23^, ^24^ first reported on the pelvic parameters, including PI, PT, and SS, increasing attention has been paid to the pelvic parameters and spinopelvic alignment in studying DLS^13^, ^25^, ^26^. PI is a constant anatomical parameter in adult subjects. There are several studies examining the influence of PI and spine–pelvic mismatch (PI‐LL) on surgical clinical outcomes^14^, ^15^. However, there are fewer studies considering the role of PI in the progress of sagittal imbalance. The present study investigates how patients with different PI maintain their sagittal balance. A normal PI mean value ranges from 50° to 55° and a normal individual PI value ranges from 28° to 84°^22^. The PI value of the patients in the present study was 52.00° ± 11.03°. These patients were divided into two groups according to their PI values. The patients’ PI value in Group 1 was smaller than 50°. The patients’ PI value in Group 2 was equal to or larger than 50°. The PI value of Group 1 was significantly smaller than the PI value of Group 2 (40.90° ± 5.81°, 58.28° ± 7.82°, *P* = 0.000). The results (Table 1) showed that there was no significant difference between the two groups in coronal characters, such as the lumbar scoliosis Cobb angle, the coronal shift angle, and the vertebra number included in the scoliosis. Although there was also no significant difference in TK, SVA, and sagittal shift angle, the LL of Group 1 was smaller than that of Group 2, and the GK was larger than that of Group 2. Because both the smaller LL and larger GK would lead to larger sagittal deviation, the results seem paradoxical; that is, that the two groups of patients with different LL and GK had similar degrees of sagittal imbalance. Considering that both the LL and GK include SS, the LL and GK was adjusted by subtracting SS. Then the statistical results showed that there was no significant difference in LL‐SS and GK‐SS between the two groups.

The spine–pelvic mismatch (PI‐LL) is one of the spinopelvic parameters and is an indicator in intraoperative planning for lumbar deformity surgery^27^, ^28^. A normal PI‐LL value ranges from −9° to +9°^14^, ^22^. Hence, the deviation degree of PI‐LL to normal value could reflect the change of LL. In this study, the PI‐LL value of these patients was 19.86° ± 10.97°, suggesting that the lumbar lordosis was significantly decreased. The results (Table 1) revealed that there was no significant difference in PI‐LL between the two groups. The Pearson test results showed that PI‐LL had significant negative correlations with TK, LL, and LL‐SS (respectively, *r* = −0.549, *P* = 0.001; *r* = −0.578, *P* = 0.000; *r* = −0.775, *P* = 0.000). PI‐LL had a positive correlation with the sagittal shift angle and SVA. This suggested that reduction of the LL was the main cause of patients’ sagittal imbalance; the decrease of TK was one of the compensatory factors for maintaining patients’ sagittal balance.

In addition, by comparing the APPA‐4 of the two groups, we found that the APPA‐4 of Group 1 was significantly larger than that of Group 2. This suggested that DLS patients with smaller PI tend to rely more on the pelvic retroversion to maintain the sagittal balance than patients with larger PI. Subjects with smaller PI generally have a smaller LL, and then need a smaller TK to maintain the sagittal balance. DLS patients with smaller TK have a lower ability to maintain the sagittal balance by decreasing TK. During the development of sagittal imbalance, for maintaining the balance position, DLS patients with smaller TK were likely to start the pelvic retroversion compensatory mechanism earlier than the patients with larger PI.

### *Limitations*

There are some limitations in the present study. First, it is a retrospective and observational study. Second, the sample size of each group was relatively small. Third, this study does not include clinical outcomes, such as the ODI.

### *Conclusion*

The APPA‐4 is reliable as degree of the pelvic sagittal retroversion. In DLS, patients with smaller PI tended to rely more on the pelvic retroversion to maintain the sagittal balance than patients with larger PI, or patients with smaller PI were likely to start up the pelvic retroversion compensatory mechanism earlier than the patients with larger PI.
